# Supplementary material for: Food Insecurity During COVID-19 in Children with End-Stage Kidney Disease: A Pilot Study
Source: Res Sq. 2022 Mar 30:rs.3.rs-910466. Preprint. [Version 1] doi: 10.21203/rs.3.rs-910466/v1 (PMC8978953; doi:10.21203/rs.3.rs-910466/v1)
Supplement: Supplement 1 [file a4aee99020ddd0fd69409cf9.docx]

**Supplemental Table 1**. Patient Characteristics Stratified by Food Security Status

|  | Food Insecure (n=18) | Food Secure (n=11) | All Participants  (n=29) |
| --- | --- | --- | --- |
| Male  Age (years), Mean (SD)  Race  American Indian/Alaska Native  Black  Caucasian  Other/Unknown  Reason for ESKD  Glomerulonephritis  Cystic kidney disease  Renal dysplasia  Obstructive uropathy  FSGS  Congenital nephrotic syndrome  Other | 12 (67%)  12.9 (4.5)  2(11%)  7 (39%)  6 (33%)  3(17%)  4 (22%)  0  5 (28%)  3 (17%)  3 (17%)  0  3 (17%) | 4 (36%)  9.4 (6.1)  0  4 (36%)  5 (45%)  2 (18%)  1 (9%)  1 (9%)  3 (27%)  2 (18%)  1 (9%)  1 (9%)  2 (18%) | 16 (55%)  11.6 (5.32)  2 (7%)  11 (38%)  11 (38%)  5 (17%)  5 (27%)  1 (3%)  8 (28%)  5 (17%)  4 (14%)  1 (3%)  5 (17%) |
| Dialysis Modality  Hemodialysis  Peritoneal Dialysis | 17 (94%)  1 (6%) | 8 (73%)  3 (27%) | 25 (86%)  4 (14%) |

COVID-19, Coronavirus Disease 19; ESKD, End-Stage Renal Disease; FSGS, Focal Segmental Glomerulosclerosis; SD, Standard Deviation.

**Supplemental Table 2**. Markers of Nutritional Status Prior to and During COVID Pandemic Among Food Insecure and Food Secure Children with ESKD

|  | Baseline | During COVID | *p* value |
| --- | --- | --- | --- |
| Food Insecure (n=18)  Phosphorus (median, IQR)  Phosphorus in goal range (percent) | 5.3 (2.1)  9 (50%) | 6.7 (2.1)  2 (11%) | 0.04  0.03 |
| Food Secure (n=11)  Phosphorus (median, IQR)  Phosphorus in goal range (percent) | 5.5 (2.7)  4 (36%) | 6.0 (2.0)  4 (36%) | 0.32  1.00 |
